# Milk Diet

**Published:** 1875-01

**Authors:** 


### MILK DIET.

The greater the advance in knowledge of the science of medicine, and of the care and treatment of the human body to preserve its health, the more importance is attached to the use of milk for food, and to its healing properties when administered in illness. Since the era of doctresses and medical women as lecturers, the laws of health are more studied in the family; the "ounce of prevention"—the road to health by proper exercise and diet, is beginning to be regarded with its due importance. Milk contains nourishment for every part of the body. Bone, muscle, brain, and flesh can be sustained in fine condition by milk diet. The human frame is healthier when fed by variety in food; yet a certain quantity of milk should be taken every day by every person. There never was any article of diet so subject to whims and ignorant prejudices as milk. Some dislike it, others it makes "sleepy," "bilious," or "headachy." It affects the bowels of many as physic, and is constipating to others. The persons who dislike milk, or with whom it "does not agree," are invariably the ones who require it, and whom it would probably rejuvenate did they so prepare it as to make it palatable and suitable to their particular constitutions. Milk diluted one-third with lime-water, will not cause any one biliousness or headache, and, if taken regularly, will so strengthen the stomach as to banish these disorders. It may be taken with acid of some kind when it does not easily digest. The idea that milk must not be eaten with pickles is not an intelligent one, as milk curdles in the stomach nearly as soon as it is swallowed. When milk is constipating, as it is frequently found to be by persons who drink freely of it in the country in summer time, a little salt sprinkled in each glassful will prevent the difficulty. When it has an opposite result, a few drops of brandy in each goblet of milk will obviate its purgative effect. As milk is so essential to the health of our bodies, it is as well to consider *when* to take it, as how. It is a mistake to drink milk between meals, or with food at the table. In the former case it will destroy the appetite, and in the latter it is never proper to drink anything. After finishing each meal a goblet of pure milk should be drank, and if any one wishes to grow fleshy, a pint taken before retiring at night will soon cover the scrawniest bones. Although nowadays we see a great many fleshy females, there are many lean and lank ones who sigh for the fashionable measure of plumpness, and who would be vastly improved in health and appearance could their figures be rounded with good, solid flesh. Nothing is more coveted by thin women than a full figure, and nothing will so rouse the ire, and provoke the scandal of one of these "clipper builds," as the consciousness of plumpness in a rival. In cases of fever and summer complaint, milk is now given with excellent results. The idea that milk is "feverish" has exploded, and it is now the physician's great reliance in bringing through typhoid patients, or those in too low a state to be nourished by solid food. It is a mistake to scrimp the milk-pitcher. Take more milk, and buy less meat. Look to your milkman, have large-sized, well-filled milk-pitchers on the table each meal, and you will also have sound flesh and light doctor's bills.

But one thing is of the first importance, viz., that the milk must be pure and sweet. Milk which comes from badly-fed cows, or which has been kept till it becomes stale, is worse than worthless as food. It will breed a terrible train of evils. There are not many stomachs which can comfortably digest milk which is twenty-four hours old. Pure, new milk, warm from the cow, if taken in moderation, will not constipate nor occasion heaviness or headache. To some persons it may induce a trifling sense of fullness at first, but that will pass off in three or four days. Thousands of cases of dyspepsia, chronic, or habitual headache and gastric disturbance may be cured by the use of pure, sweet milk as the sole beverage and the chief food.

